# Comparison of Xpert MTB/RIF Assay and the Conventional Sputum Microscopy in Detecting *Mycobacterium tuberculosis* in Northern Thailand

**DOI:** 10.1155/2015/571782

**Published:** 2015-04-30

**Authors:** Kanokwan Pinyopornpanish, Romanee Chaiwarith, Chansom Pantip, Rassamee Keawvichit, Kanlaya Wongworapat, Phadungkiat Khamnoi, Khuanchai Supparatpinyo, Thira Sirisanthana

**Affiliations:** ^1^Department of Medicine, Faculty of Medicine, Chiang Mai University, Muang, Chiang Mai 50200, Thailand; ^2^Research Institute for Health Sciences, Faculty of Medicine, Chiang Mai University, Muang, Chiang Mai 50200, Thailand; ^3^Central Diagnostic Laboratory, Chiang Mai University Hospital, Muang, Chiang Mai 50200, Thailand

## Abstract

*Background.* Despite low sensitivity in detection of *Mycobacterium tuberculosis*, sputum acid-fast smear remains the main diagnostic method. This study aimed to compare the diagnostic performance of Xpert MTB/RIF assay versus conventional sputum acid-fast smear. *Materials and Methods.* A cross-sectional study was conducted at Chiang Mai University Hospital, Thailand. Patients who were ≥15 years old and had clinically suspected pulmonary tuberculosis were included. *Results.* 109 specimens from 57 patients were included. Using MGIT sputum culture as a reference standard, the sensitivity (SEN) and specificity (SPEC) for Xpert were 95.3% (95% CI, 84.2%, 99.4%) and 86.4% (95% CI, 75.7%, 93.6%). The SEN and SPEC for sputum acid-fast smear were 60.5% (95% CI, 44.4%, 75.0%) and 98.5% (95% CI, 91.8%, 100%). Xpert had significantly higher sensitivity (*p* value < 0.001) and lower specificity (*p* value = 0.022) than sputum acid-fast smear. Among 43 culture-proven *M. tuberculosis* specimens, sensitivity of Xpert was 100% (95% CI, 86.7%, 100%) in acid-fast positive smears (*n* = 26) and 88.2% (95% CI, 63.5%, 98.5%) in acid-fast negative smears (*n* = 17). *Conclusions.* The good sensitivity and specificity of Xpert assay in detecting *M. tuberculosis* from sputum specimens may help in early diagnosis and treatment of pulmonary tuberculosis, particularly among patients who had acid-fast negative sputum smear.

## 1. Introduction

Tuberculosis (TB) is a major health problem across the world. Thailand is one of the high TB burden countries. In 2012, World Health Organization (WHO) estimated that the incidence of TB in Thailand was 119 cases per 100,000 populations with 9,200 annual deaths. Among all TB cases, 12,000 were infected with HIV, and there were 2,200 annual deaths [1]. Early diagnosis and prompt treatment of TB are crucial to reduce morbidity and mortality, secondary drug resistance, and transmission of TB.

Despite low sensitivity in detection of* Mycobacterium tuberculosis*, acid-fast sputum smear remains the main diagnostic method in most countries, especially in resource-limited settings [2]. In HIV infected patients with pulmonary TB, 24–61% have acid-fast negative sputum smear [3]. Mycobacterial culture is the gold standard and the most sensitive method for TB diagnosis; however, the use in clinical practice is limited due to a slow turnaround time, biosafety requirements, and high cost [4, 5].

Recently, in 2011, WHO endorsed the wide use of Xpert MTB/RIF assay, a fully automated diagnostic molecular test using real-time polymerase chain reaction (PCR) technology to simultaneously detect* M. tuberculosis* and rifampicin resistance mutations in the rpoB gene. This assay can provide the results within 2 hours [6]. Several studies have demonstrated that Xpert assay is highly sensitive and specific in diagnosis of both pulmonary [7, 8] and extrapulmonary TB [9, 10]. Furthermore, Xpert assay was shown to be cost-effective for TB diagnosis, compared to microscopy in low and middle income settings [11]. Therefore, Xpert assay is strongly recommended as the initial diagnostic test in individuals suspected of having multidrug resistant (MDR) TB and in those with HIV/TB coinfection. It is also recommended as a follow-on test in TB-suspected patients with acid-fast negative sputum smear.

However, Xpert assay is not widely recognized as a diagnostic test for TB in Thailand. This study, therefore, was conducted to evaluate diagnostic performance of Xpert assay in northern Thai patients with clinically suspected pulmonary tuberculosis, using tuberculosis culture as a reference standard.

## 2. Materials and Methods

### 2.1. Study Design and Population

We conducted a cross-sectional study at Chiang Mai University Hospital, Chiang Mai, Thailand, between September 2012 and November 2013. The inclusion criteria were male or female patients with: (1) age ≥15 years; (2) clinically suspected pulmonary tuberculosis, which was defined as having 2 or more of the following symptoms: fever, chronic cough, weight loss, pleuritic chest pain, hemoptysis, and with or without abnormal chest radiograph compatible with pulmonary tuberculosis (cavitary lesion, infiltration, and miliary pattern); and (3) no history of receiving antituberculous drug within 3 months before enrollment.

### 2.2. Specimen Collection and Processing

The eligible patients were asked to provide at least 1 expectorated sputum specimen to the maximum of 3 specimens. Sputum acid-fast smear was performed on fresh specimen at the Central Diagnostic Laboratory, Chiang Mai University Hospital, Faculty of Medicine. The specimen was then decontaminated and separated into 2 samples which were blindly tested at 2 laboratory sites.

- Liquid-media mycobacterial culture of all sputum specimens using Mycobacteria Growth Indicator Tube (MGIT) method as a reference standard was performed at the Central Diagnostic Laboratory, Chiang Mai University Hospital.
- Xpert MTB/RIF assay (Cepheid) was performed at Mycobacterial Laboratory, Research Institute for Health Sciences (RIHES), Chiang Mai University. The method was performed according to manufacturer's instruction of Xpert MTB/RIF [12].

The specimen was excluded from the analysis if (1) it was an invalid sample for Xpert assay or sample error according to Cepheid package insert, or (2) the culture was contaminated.

### 2.3. Data Collection

Demographic data including age, sex, past medical history of lung diseases, and HIV serostatus were recorded. Clinical data including fever, days of fever, chest pain, dyspnea, hemoptysis, weight loss, cough, and the extrapulmonary sites of tuberculosis were collected. Laboratory results included sputum acid-fast smear, sputum mycobacterial culture results, sputum Xpert MTB/RIF results, and chest radiographic findings were collected. Sputum acid-fast smear results were categorized into positive (reported AFB found per field as 1+, 2+, 3+, and 4+) or negative. Sputum Xpert MTB/RIF results were also categorized into positive (reported Xpert detected as very low, low, medium, and high), or negative. Sputum cultures were reported as* M. tuberculosis*, NTM, contaminated culture, or no growth.

### 2.4. Statistical Analysis

Descriptive data were presented in percentages, mean ± SD, and median (IQR) as appropriate. The sensitivity, specificity, positive predictive value (PPV), and negative predictive value (NPV) of Xpert and sputum acid-fast smear were compared to tuberculosis culture as a reference standard using contingency 2 × 2 tables and exact binomial confidence intervals. The sensitivity and specificity between sputum smear and Xpert were compared using McNemar's test. Among culture-confirmed pulmonary tuberculosis, comparison of characteristics between patients with positive and negative sputum acid-fast smear was performed using *χ* ^2^ test or Fisher's exact test for categorical data and Student's *t*-test or Mann-Whitney *U* test for continuous data. Factors associated with negative sputum acid-fast smear were tested in univariate models. Factors with the *p* value of <0.10 from univariate analysis were then tested in a multivariate logistic regression model.

All statistical analyses were performed using Stata statistical software version 10.0 (Stata Statistical Software: Release 10.0, Stata Corporation, College Station, TX, 2007).

This study was approved from the Faculty of Medicine, Chiang Mai University ethical committee.

## 3. Results

### 3.1. Demographic and Clinical Data

Sixty consecutive patients who met the inclusion criteria providing 127 sputum specimens were enrolled to the study. Of 127 specimens, 2 were invalid for Xpert, 2 were sample error, 14 were contaminated, and 11 grew NTM. After exclusion of 18 specimens, 109 sputum specimens from 57 patients were included in the analysis. Twenty-nine, 15, and 12 patients provided 1, 2, and 3 specimens, respectively.

Demographic data of 57 patients are shown in Table 1. Twenty-three patients (40.4%) were female and the mean age was 55.6 ± 20.1 years. Thirteen patients (22.8%) had underlying lung diseases and 15 patients (26.3%) had HIV infection.

### 3.2. Identification of* M. tuberculosis* and Drug Susceptibility Test

Among 109 sputum specimens,* M. tuberculosis* was isolated by MGIT culture from 43 specimens (39.4%). Xpert assay and sputum acid-fast smear yielded positive result in 46 specimens (45.9%) and 27 specimens (24.8%), respectively. The median time to get the culture results was 45 days (IQR 42, 57 days), whereas the median time to get results from Xpert assay and sputum acid-fast smear was 6 days (IQR 3, 8 days) and 1 day, respectively.

From MGIT culture, 3 specimens from 1 patient showed INH resistance, 4 specimens from 2 patients showed streptomycin resistance, and 3 specimens from 1 patient showed both INH and rifampin resistance. Xpert assay failed to detect rifampin resistance in all 3 specimens with rifampin resistance.

### 3.3. Performance of Xpert Assay and Sputum Acid-Fast Smear

Table 2(a) shows the two by two contingency table comparing between Xpert assay and sputum culture. Using sputum culture as a reference standard, the overall sensitivity, specificity, PPV, and NPV for Xpert assay were 95.3%, 86.4%, 82.0%, and 96.6%, respectively.

Table 2(b) shows the two by two contingency table comparing between sputum acid-fast smear and sputum culture. Using sputum culture as a reference standard, the overall sensitivity, specificity, PPV, and NPV for sputum acid-fast smear were 60.5%, 98.5%, 96.3%, and 79.3%, respectively.

The sensitivity, specificity, PPV, and NPV and their correspondent 95% CIs are shown in Table 3. Xpert assay had statistically significant higher sensitivity than sputum acid-fast smear (*p* value < 0.001), whereas the specificity was statistically lower than sputum acid-fast smear (*p* value 0.022).

Among 43 culture-proven* M. tuberculosis* specimens, sensitivity of Xpert assay was 100% (95% CI, 86.7%, 100%) among smear positive specimens (*n* = 26) and 88.2% (95% CI, 63.5%, 98.5%) among smear negative specimens (*n* = 17).

Xpert assay, by subgroup analysis in HIV-infected patients (29 specimens), had sensitivity of 100%, specificity of 100.0%, PPV of 76.9%, and NPV of 100% (Table 3).

### 3.4. Characteristics among Culture-Confirmed Pulmonary Tuberculosis Patients

Among 27 patients with culture-confirmed pulmonary tuberculosis, sputum acid-fast smear was positive in 18 patients (66.7%).  Table 4 shows the comparison of demographic and clinical characteristics between patients with positive and negative sputum acid-fast smear. We found that there was no significant factor of likelihood for negative sputum acid-fast smear from both univariate and multivariate analyses.

## 4. Discussion

A recent meta-analysis that included 9,557 participants from 27 studies showed that Xpert assay of respiratory specimens had a pooled sensitivity of 89% (95% CI 85%, 92%) and specificity of 99% (95% CI 98%-99%) in the diagnosis of pulmonary tuberculosis [13]. In our study that also used respiratory specimens, the overall sensitivity of Xpert assay was 95.3% and specificity was 86.4%, which were slightly higher and lower than those previous studies, respectively. Compared to conventional sputum acid-fast smear that had sensitivity of 60.5% and specificity of 98.5%, our study found that Xpert assay had statistically significant higher sensitivity (*p* value < 0.001) and lower specificity (*p* value 0.022).

Data from several studies in culture-proven TB patients with acid-fast positive sputum specimens showed that Xpert assay had a pooled sensitivity of 98% (95% CI 97%, 99%) in the detection of* M. tuberculosis* [12]. Our study, although with a small number of samples, also found a good concordant result with those studies; the sensitivity of Xpert assay to detect* M. tuberculosis* was 100% in acid-fast positive sputum specimens. This finding confirmed that it is unlikely for Xpert assay to miss the diagnosis of TB in patients with positive sputum acid-fast smear. However, our study showed that the sensitivity of Xpert assay for detection of* M. tuberculosis* in culture-proven TB specimens but negative acid-fast smear was 88.2%. Our study showed considerably higher sensitivity than those in previous studies that had a pooled sensitivity of 67% (95% CI 60%, 74%) with a range from 57.1 to 76.9% [13]. Our study also showed a 100% sensitivity of Xpert assay in detecting* M. tuberculosis* in subgroup of 29 HIV infected patients; this was higher than 84% and 69.6% in previous studies [14, 15]. The difference in sensitivity of Xpert assay on detection of* M. tuberculosis* among studies may be resulted from the difference in inclusion criteria. Inclusion of patients with minimal symptoms or normal chest X-ray that may have low bacillary load could result in higher rate of negative results from Xpert assay. In addition, the technique to obtain sputum specimens varied among studies. Sputum induction may cause less bacillary load compared to the expectorated sputum [12, 16].

Although WHO endorsed the wide use of Xpert MTB/RIF assay, this test is expensive and available only in large medical centers in Thailand. Using this assay in all patients with clinically suspected tuberculosis may neither be possible nor cost-effective in resource-limited settings in particular where the laboratory technicians have high experience in microscopy. As mentioned above, our study and other studies all confirm that positive acid-fast smear correlates well with Xpert assay and TB culture; patients with positive acid-fast smear may not have benefit from Xpert assay in detecting TB but may have benefit in detecting rifampin resistance if it exists. In order to identify predicting factors for having negative sputum acid-fast smear, we compared demographic and clinical characteristics among patients with culture-confirmed pulmonary tuberculosis who had positive and negative sputum acid-fast smears. Patient who has one of these factors, if any, and has negative sputum acid-fast smear may have benefit from Xpert assay. Unfortunately, we could not identify any factors associated with negative sputum acid-fast smear in patients with tuberculosis. Failure to demonstrate that may be due to small number of patients in this study and further studies with larger sample size may be warranted.

Our study demonstrated the overall specificity of 86.4% (75.7–93.6%), which was slightly lower than those in previous studies that had a range from 94% to 100% [12]. Five sputum specimens from 4 patients in our study had positive Xpert assay but negative TB culture and negative acid-fast smear. One patient with HIV infection had cervical lymphadenopathy and* M. tuberculosis* was isolated from his lymph node culture. Two patients had other sputum specimens that were acid-fast positive but cultures were not performed. This suggested that MGIT culture may not be a good reference standard. Using the reference standard with low sensitivity could lead to a lower specificity of the test. In fact, Xpert assay may be more appropriate for reference standard since previous analytical study demonstrated that MGIT culture can detect* M. tuberculosis* at the level of 5 × 10^5^ cfu/mL [17] while the Xpert assay can detect* M. tuberculosis* at the level of as low as 131 cfu/mL or 5 genome copies of DNA [6, 18].

Interestingly, 10 samples from 6 patients in our study grew NTM. Four samples from 3 patients were weakly positive by Xpert assay. In one patient who produced 2 sputum specimens, one specimen grew* M. tuberculosis* and the other grew NTM. We, therefore, believe that the positive result in these 4 specimens may be from infection of both NTM and* M. tuberculosis* in the same patients. Data from previous studies also confirm that it is not common for Xpert assay to produce a false positive result in NTM growth specimens [13]. Recent analytical study also found that Xpert assay can correctly detect* M. tuberculosis* in patients who had mixed infection with high number of NTM and low number of* M. tuberculosis* [19].

Of note, invalid and error results from Xpert assay occurred in 4 of 127 specimens (3.1%) whereas contaminated culture occurred in 14 of 127 specimens (11.0%). In addition, median turnaround time from sputum collection to get Xpert results was faster than from culture results. Due to the time-taking process to prepare and run the test for each specimen, we decide to run the test once we have four samples ready instead of one at a time. Therefore, the median turnaround time for Xpert assay in our study was 6 days, comparing to 45 days for culture results. These demonstrated that Xpert assay was more reliable than sputum culture for* M. tuberculosis* and had more rapid turnaround time.

Our study has several limitations. First, there was a high rate of contaminated culture specimens (11.0%). This may indicated poor technique of specimen collection and decontamination. Second, the relatively small numbers of participants limited the study power. Finally, since there were only 3 specimens from one patient with rifampin resistance in this study, data was not sufficient to evaluate Xpert performance in detecting rifampin resistance.

## 5. Conclusions

This study demonstrated good sensitivity and specificity of Xpert assay in detecting* M. tuberculosis* from respiratory specimens. This is particularly helpful in making a rapid diagnosis and initiating prompt treatment of tuberculosis in patients who had negative sputum acid-fast smear.

## Figures and Tables

**Table 1:** Demographic data of 57 patients with clinically suspected pulmonary tuberculosis.

Demographic data	Patients (*n* = 57)
Female	23 (40.4%)
Age (mean ± SD)	55.6 ± 20.1
Underlying lung diseases	
Chronic obstructive pulmonary disease (COPD)	6 (10.7%)
Bronchiectasis	4 (7.1%)
Other chronic lung diseases	3 (5.4%)
HIV serostatus	
Positive	15 (26.3%)
Negative	40 (71.9%)
Not tested	1 (1.8%)

**(a):** 

	Positive Xpert assay	Negative Xpert assay	Total
Culture grew *M*. *tuberculosis *	41	2	43
Culture grew NTM or no growth	9	57	66

Total	50	59	109

NTM: nontuberculous mycobacteria, 4 in positive Xpert assay and 6 in negative Xpert assay.

**(b):** 

	Positive sputum acid-fast smear	Negative sputum acid-fast smear	Total
Culture grew *M*. *tuberculosis *	26	17	43
Culture grew NTM or no growth	1	65	66

Total	27	82	109

NTM: nontuberculous mycobacteria, 10 in negative sputum acid-fast smear.

**Table 3:** Sensitivity, specificity, positive predictive value, and negative predictive value of Xpert assay and sputum acid-fast smear comparing to sputum culture.

	Xpert assay	95% confidence interval	Acid-fast smear	95% confidence interval
Specimens from all patients (*n* = 109)				
SEN	41/43 = 95.3%	84.2–99.4%	26/43 = 60.5%	44.4–75.0%
SPEC	57/66 = 86.4%	75.7–93.6%	65/66 = 98.5%	91.8–100%
PPV	41/50 = 82.0%	68.6–91.4%	26/27 = 96.3%	81.0–99.9%
NPV	57/59 = 96.6%	88.3–99.6%	65/82 = 79.3%	68.9–87.4%

Specimens from HIV-infected patients (*n* = 29)				
SEN	10/10 = 100%	69.2–100%	6/10 = 60%	26.2–87.8%
SPEC	16/16 = 100%	79.4–100%	19/19 = 100%	82.4–100%
PPV	10/13 = 76.9%	46.2–95.0%	6/6 = 100%	54.1–100%
NPV	16/16 = 100%	79.4–100%	19/23 = 80%	61.2–95.0%

Specimens from HIV-uninfected patients (*n* = 77)				
SEN	28/30 = 93.3%	77.9–99.2%	20/30 = 66.7%	47.2–82.7%
SPEC	41/43 = 95.3%	84.2–99.4%	46/47 = 97.9%	88.7–99.9%
PPV	28/34 = 82.4%	65.5–93.2%	20/21 = 95.2%	76.2–99.9%
NPV	41/43 = 95.3%	84.2–99.4%	46/56 = 82.1%	69.6–91.1%

SEN: sensitivity; SPEC: specificity; PPV: positive predictive value; NPV: negative predictive value.

**Table 4:** Demographic and clinical characteristics of 27 TB culture-proven patients with positive and negative sputum acid-fast smears.

Characteristic	Positive acid-fast smear (*n* = 18)	Negative acid-fast smear (*n* = 9)	*p* value
Female	5 (27.8)	6 (66.7)	0.097
Age	48.3 (±17.2)	61.7 (±22.8)	0.136
HIV infection	13 (72.2)	7 (77.8)	0.301
Presence of underlying lung disease	5 (27.8)	2 (22.2)	0.395
Presence of fever	14 (77.8)	4 (44.4)	0.083
Duration of illness >7 days	8 (44.4)	4 (44.4)	1.000
Presence of chest pain	3 (16.7)	2 (22.2)	0.726
Presence of dyspnea	4 (22.2)	1 (11.1)	0.636
Presence of cough	17 (94.4)	6 (66.6)	0.093
Hemoptysis	1 (5.6)	1 (11.1)	1.000
Significant weight loss	5 (27.8)	2 (22.2)	0.263
Concurrent extrapulmonary infection	1 (5.6)	2 (22.2)	0.250
Abnormal CXR	17 (94.4)	9 (100)	0.694

Data are presented in number (%), or mean ± SD.
